# miR‐541 suppresses proliferation and invasion of squamous cell lung carcinoma cell lines via directly targeting high‐mobility group AT‐hook 2

**DOI:** 10.1002/cam4.1491

**Published:** 2018-04-16

**Authors:** Li Xu, Bin Du, Qi‐Jue Lu, Xiao‐Wen Fan, Ke Tang, Lie Yang, Wei‐Lin Liao

**Affiliations:** ^1^ Department of Thoracic Surgery Shanghai Pulmonary Hospital Tongji University School of Medicine Shanghai 200433 China; ^2^ Department of Thoracic Surgery Chengdu Military General Hospital Chengdu 610083 Sichuan China; ^3^ Department of Thoracic Surgery Changhai Hospital Second Military Medical University Shanghai 200438 China

**Keywords:** HMGA2, invasion, migration, miR‐541, proliferation, squamous cell lung carcinoma

## Abstract

An increasing number of studies have demonstrated that micro‐ribonucleic acids (miRNAs) are important tumor suppressors during carcinogenesis. However, the function of miRNA‐541 (miR‐541) in malignancies, especially lung cancer, has not been widely reported. In this study, miR‐541 expression was significantly decreased in squamous cell lung carcinoma (SCLC) cancerous tissue and SCLC cell lines. To analyze miR‐541 function in SCLC, we overexpressed miR‐541 in SCLC cell lines (SK‐MES‐1 and H226). According to the CCK8, wound scratch, and transwell invasion assay results, miR‐541 overexpression significantly inhibited SCLC cell proliferation, migration, and invasion ability. Next, using RT‐PCR, Western blotting, immunocytochemistry, and luciferase assays, HMGA2 was identified, for the first time, as a direct regulatory target of miR‐541 in SK‐MES‐1 and H226 cells. Furthermore, upregulating HMGA2 expression significantly alleviated the suppressive effects of miR‐541 on SK‐MES‐1 and H226 cell proliferation, migration, and invasion. In summary, our study revealed that miR‐541 inhibited SCLC proliferation and invasion by directly targeting HMGA2.

## Introduction

Lung cancer, a leading cause of tumor‐associated death worldwide, is the most common malignant tumor 1. Non‐small‐cell lung cancer (NSCLC), a large class of lung cancer, causes over 80% of lung cancer‐related deaths 2. In China, squamous cell lung carcinoma (SCLC) is the second most common pathological NSCLC subtype 3. Despite the great improvements in comprehensive therapies, including surgical treatment, chemotherapy, and molecular‐targeted treatments, that have been made in recent years for squamous cell lung carcinoma (SCLC), the prognosis of this disease remains unsatisfactory due to tumor recurrence and metastasis, which are great challenges for clinical SCLC treatment. As a result, to develop new therapeutic strategies for SCLC, understanding SCLC progression and identifying novel endogenous molecules that suppress that progression is of critical importance.

Micro‐ribonucleic acids (miRNAs) are a class of endogenous small noncoding RNAs. miRNAs act as novel mediators during organism development and human disease via posttranscriptionally regulating the related target genes 4. Abnormal miRNA expression is related to cancer. Different types of cancers have different miRNA expression patterns 5. An increasing amount of evidence shows that miRNAs regulate various biological processes during tumorigenesis and metastasis 6, 7, 8. In addition, specific miRNAs inhibit carcinogenesis progression 9, 10, 11.

One of our recent studies showed that HMGA2, an acknowledged oncogene in other malignancies 12, is highly overexpressed and enhances cell proliferation and invasion in SCLC 13. However, relatively few studies have investigated the regulation of HMGA2 during SCLC progression. In this study, we found that miR‐541 expression, which is decreased in SCLC, inhibited SCLC via directly negatively regulating HMGA2 levels, which enriched the HMGA2 regulatory network during SCLC progression.

## Materials and Methods

### Tissue specimen collection

For qRT‐PCR analysis, cancerous and adjacent normal tissue specimens were obtained from 15 patients with peripheral SCLC who underwent video‐assisted thoracic surgery (VATS) lobectomies and lymph‐node dissection at the Department of Thoracic Surgery, Chengdu Military General Hospital, from August to December 2016. All cancerous and matching adjacent normal tissue samples used in this study were initially histologically diagnosed by the Department of Pathology, Chengdu Military General Hospital.

### Cell culture

The human SCLC SK‐MES‐1 and H226 cell lines were obtained and cultured as previously described 13. BEAS‐2B human bronchial epithelial cells obtained from Shanghai Cell Bank, Shanghai, China, were cultured in DMEM supplemented with 10% fetal bovine serum, 100 *μ*g/mL streptomycin, and 100 units/mL penicillin at 37°C in a 5% CO_2_ atmosphere.

### Quantitative real‐time reverse‐transcription polymerase chain reaction (qRT‐PCR)

Total RNA from fresh tissue specimens and cells was extracted, and cDNA was synthesized using a One Step PrimeScript^®^ miRNA cDNA Synthesis kit (D350A; TaKaRa Bio) for miRNA and a PrimeScript RT Reagent kit (TakaRa Bio, 19 Northeast 2nd street, Dalian, China) for mRNA. SYBR Premix EX Taq (TaKaRa Bio) was used for quantitative real‐time polymerase chain reaction, which was performed using a LightCycler^®^ 480 Real‐Time PCR System (F. Hoffmann‐La Roche AG, Basel, Switzerland). The primers used in the qRT‐PCR assays were as follows: miR‐541‐3p forward 5′‐GGGTGGTGGGCACAGAATC‐3′ and reverse 5′‐CAGTGCGTGTCGTGGAGT‐3′; U6 snRNA forward 5′‐CTCGCTTCGGCAGCACA‐3′ and reverse 5′‐AACGCTTCACGAATTTGCGT‐3′; HMGA2 forward 5′‐AGCAGCAGCAAGAACCAACC‐3′ and reverse 5′‐CCTGAGCAGGCTTCTTCTGA‐3′; and 18s rRNA forward 5′‐CGGACACGGACAGGATTGAC‐3′ and reverse 5′‐GCATGCCAGAGTCTCGTTCG‐3′. U6 snRNA and 18s rRNA served as the internal controls. The 2^−ΔΔCT^ method was used to deal with the raw data to show the ratio results. The miR‐541 expression levels in the SCLC tissues were normalized to those in the SK‐MES‐1 cells. To statistically compare the differences between groups, log_2_ transformation was applied to deal with the ratio results to obtain the normally distributed data.

### Western blot

Western blot analyses were performed as previously described 14, with modifications. A nuclear and cytoplasmic extraction kit (CW0199S; CoWin Bioscience, Peking, China) was used to extract nuclear and cytoplasmic SCLC cell proteins, separately. Next, the nuclear protein extract from each cell group was loaded onto and run on 12% SDS polyacrylamide gels (30 *μ*g/well) and transferred onto nitrocellulose membranes (LC2000 for HMGA2 and LC2001 for lamin B_2_; ThermoFisher Scientific, 27 Xinjinqiao Road, Shanghai, China). The blots were blocked with blocking buffer (P0023B; Beyotime Biotechnology, Shanghai, China) and incubated overnight at 4°C with primary antibody, followed by incubation with the secondary antibody. Primary (P0023A; Beyotime Biotechnology) and secondary (P0023D; Beyotime Biotechnology) antibody dilution buffers were used to dilute the corresponding antibodies. The primary antibodies HMGA2 (1:500, ab52039; Abcam, 330 Cambridge Science Park, Cambridge, United Kingdom) and lamin B2 (1:1000, ab151735; Abcam) were used, with lamin B2 serving as the internal control. The secondary antibody peroxidase‐AffiniPure goat anti‐rabbit IgG (H+L) (1:5000, 111035003; Jackson ImmunoResearch, 872 West Baltimore Pike, West Grove, PA) was used. Proteins were visualized by a luminescent image analyzer (ImageQuant LAS4000; GE Healthcare Bio‐Sciences AB).

### miR‐541 and HMGA2 overexpression

The miR‐541‐mimics (B02001) and miR‐541‐scramble were synthesized by GenePharma (Shanghai, China). After the SK‐MES‐1 and H226 cells entered the logarithmic phase, the miR‐541 and miR‐541‐scramble transfections were conducted according to the Lipofectamine RNAiMAX (Invitrogen, Carlsbad, CA) instruction. The sequences of miR‐541‐mimics were as follows: sense strand: 5′‐UGGUGGGCACAGAAUCUGGACU‐3′; and antisense strand: 5′‐UCCAGAUUCUGUGCCCACCAUU‐3′. The sequences of miR‐541‐scramble were as follows: sense strand: 5′‐UUCUCCGAACGUGUCACGUTT‐3′; and antisense strand: 5′‐ACGUGACACGUUCGGAGAATT‐3′. A lentivirus vector expressing HMGA2 (lenti‐HMGA2, GOCL3651094131) and a control lentivirus vector (lenti‐control) were purchased from GeneChem Co., Ltd. (Shanghai, China).

### miR‐541 inhibition

The miR‐541 inhibitor (B03001) and negative control inhibitor were also synthesized by GenePharma (Shanghai, China). The miR‐541 inhibitor and negative control inhibitor transfections were also conducted according to the Lipofectamine RNAiMAX instruction after the BEAS‐2B cells entered the logarithmic phase. The sequence of miR‐541 inhibitor was as follows: 5′‐AGUCCAGAUUCUGUGCCCACCA‐3′. The sequence of negative control inhibitor was as follows: 5′‐CAGUACUUUUGUGUAGUACAA‐3′.

### Immunocytochemistry

SCLC and BEAS‐2B cells were seeded onto coverslips in a 24‐well plate. Paraformaldehyde was used to fix the SCLC and BEAS‐2B cells. Triton X‐100 was used to prepare the membrane. After being rinsed with PBS, the cells were blocked with 5% goat serum for 30 minutes and incubated overnight at 4°C with the primary antibody, followed by incubation with the secondary antibody. Nuclei were counterstained with DAPI. The primary antibody used was anti‐HMGA2 (1:100, ab52039; Abcam). The secondary antibody was Alexa Fluor 488 AffiniPure goat anti‐rabbit IgG (H+L) (1:400, 111545003; Jackson ImmunoResearch).

### Cell proliferation

Cell proliferation assays were performed as previously described 13, with modifications. Eight hours after transfection, the cells were plated into a 96‐well culture dish at a density of 2500 cells/well. Then, cells were incubated for 0, 24, 48, and 72 h, separately. Next, 10 *μ*L of CCK8 solution was added to each well, followed by incubation at 37°C for 1 h. Then, the absorbance of each well at 450 nm was measured.

### Cell migration

The cell migration capacity was evaluated by wound scratch assays, which were performed as previously described 15, with modifications. Twelve hours after transfection, a sterile plastic pipette tip was used to make a straight‐line scratch on the cell monolayer. For the SK‐MES‐1 cells, pictures were taken at 0 and 48 h, while for the H226 cells, pictures were taken at 0 and 24 h. The migration distance was calculated as the difference in the gap width between the two photographic time points.

### Invasion assay

The transwell invasion assay was performed as previously described 13, with modifications. Transwell insert chambers with an 8 *μ*m pore size (Corning) were used to examine the cell invasion ability. Before 1 × 10^5^ cells were seeded in the serum‐free medium in the upper chamber, the transwell filter inserts were coated with Matrigel. After the cells were incubated at 37°C for 24 h, the cells in the upper chamber were removed with a cotton swab. However, the cells that had traversed the membrane were fixed, stained, and counted.

### Luciferase assay

Lipofectamine 2000 (Invitrogen) was used to cotransfect 293T cells with pairwise combinations of psiCHECK‐HGMA2‐wild‐type (WT), miR‐541‐mimics, psiCHECK‐HMGA2‐mutated (MU), and miR‐541‐scramble. The transfection was conducted following the instructions. At 48 h after transfection, the 293T cells were lysed. The luciferase activity was measured using the Dual‐Glo Luciferase assay system (Promega) and was normalized to Renilla luciferase activity. The psiCHECK‐HGMA2‐WT plasmid containing the HMGA2 mRNA 3′ UTR was constructed by PCR cloning of chemically synthesized DNA fragments, using the following primers: forward 5′‐CCGCTCGAGCGGGGGGCGCCAACGTTCGATTTCT‐3′ and reverse 5′‐CTGTTTTGACCAAACTTTATTAATAGTTTAGCGGCCGCATTCTTAT‐3′. The underlined sequences indicate the restriction enzyme sites for Xhol and Notl, respectively. The psiCHECK‐HMGA2‐MU plasmid containing site‐directed mutations was generated using the following primers: forward 5′‐AAAAAAGGGGGGGGCAATCTCTCGGCCTGTCTTTCTCTCTCTCTCTTCCTC‐3′ and reverse 5′‐GAGGAAGAGAGAGAGAGAAAGACAGGCCGAGAGATTGCCCCCCCCTTTTTT‐3′. The underlined sequences indicate the mutated sites.

### Statistical analysis

Data are presented as the means ± SD. Statistical comparisons were analyzed with SAS software, version 9.4 (SAS Institute, Cary, NC). The quantitative data were first evaluated for normal distribution by the Shapiro–Wilk test. Next, Welch's *t*‐test was applied to compare the differences between groups. All *P*‐values are two‐sided, and a value less than 0.05 was considered to indicate a significant difference.

## Results

### miR‐541 expression is decreased in squamous cell lung carcinoma

**Figure 1:**
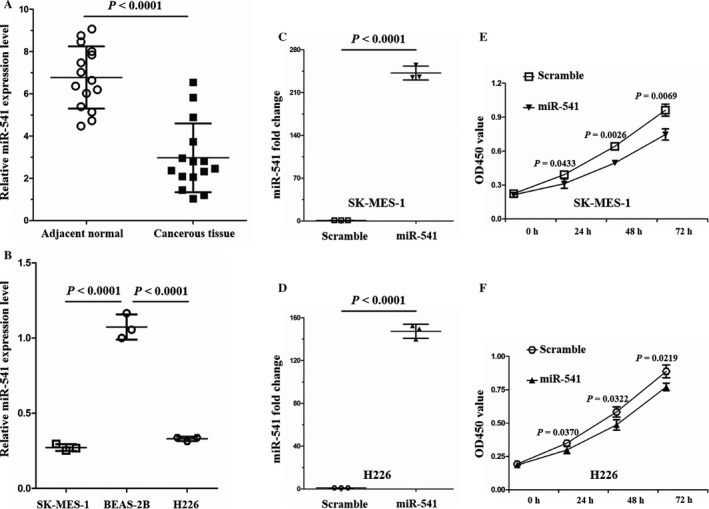
Relative miR‐541 expression levels in SCLC tissue samples and SCLC cell lines, and inhibition of SCLC cell proliferation by miR‐541 overexpression. (A) According to the qRT‐PCR results, the miR‐541 expression levels were significantly lower in the cancerous tissues (*n* = 15) than in the adjacent normal tissues (*n* = 15) of patients with SCLC. Every test was carried out in triplicate, and *n* = 3 for each tissue sample. (B) The miR‐541 expression levels were lower in the SCLC cells than in the normal human bronchial epithelial cells (*n* = 3 for each cell line). (C) Successful miR‐541 overexpression in SK‐MES‐1 cells induced by miR‐541‐mimics. Cells transfected with miR‐541‐scramble served as the control cells (*n* = 3 for each cell group). (D) Verification of miR‐541 overexpression in H226 cells. Cells transfected with miR‐541‐scramble served as the control cells (*n* = 3 for each cell group). (E, F) The OD450 values were lower in the miR‐541‐overexpressing SK‐MES‐1 and H226 cells than in the control cells at 24, 48, and 72 h (*n* = 3 for each cell group). OD450, optical density at 450 nm.

miR‐541 has been reported to be involved in several tumors 16, 17. Recently, a study revealed the potential tumor‐suppressing function of miR‐541 in lung cancer 18. To investigate the role of miR‐541 in SCLC, we first examined the miR‐541 expression levels in adjacent normal tissue and cancerous tissue from patients with SCLC. The qRT‐PCR results revealed that the miR‐541 expression levels were significantly lower in the cancerous tissue than in the adjacent normal tissue (Fig. 1A). Next, the miR‐541 expression levels in SCLC cells (SK‐MES‐1 and H226) and normal human bronchial epithelial cells BEAS‐2B were examined. The experimental results revealed that the miR‐541 expression levels were significantly lower in the SK‐MES‐1 and H226 than in the BEAS‐2B cells (Fig. 1B). Taken together, these results indicate that miR‐541 might participate in SCLC pathogenesis.

### miR‐541 suppresses SCLC cell proliferation and invasion

**Figure 2:**
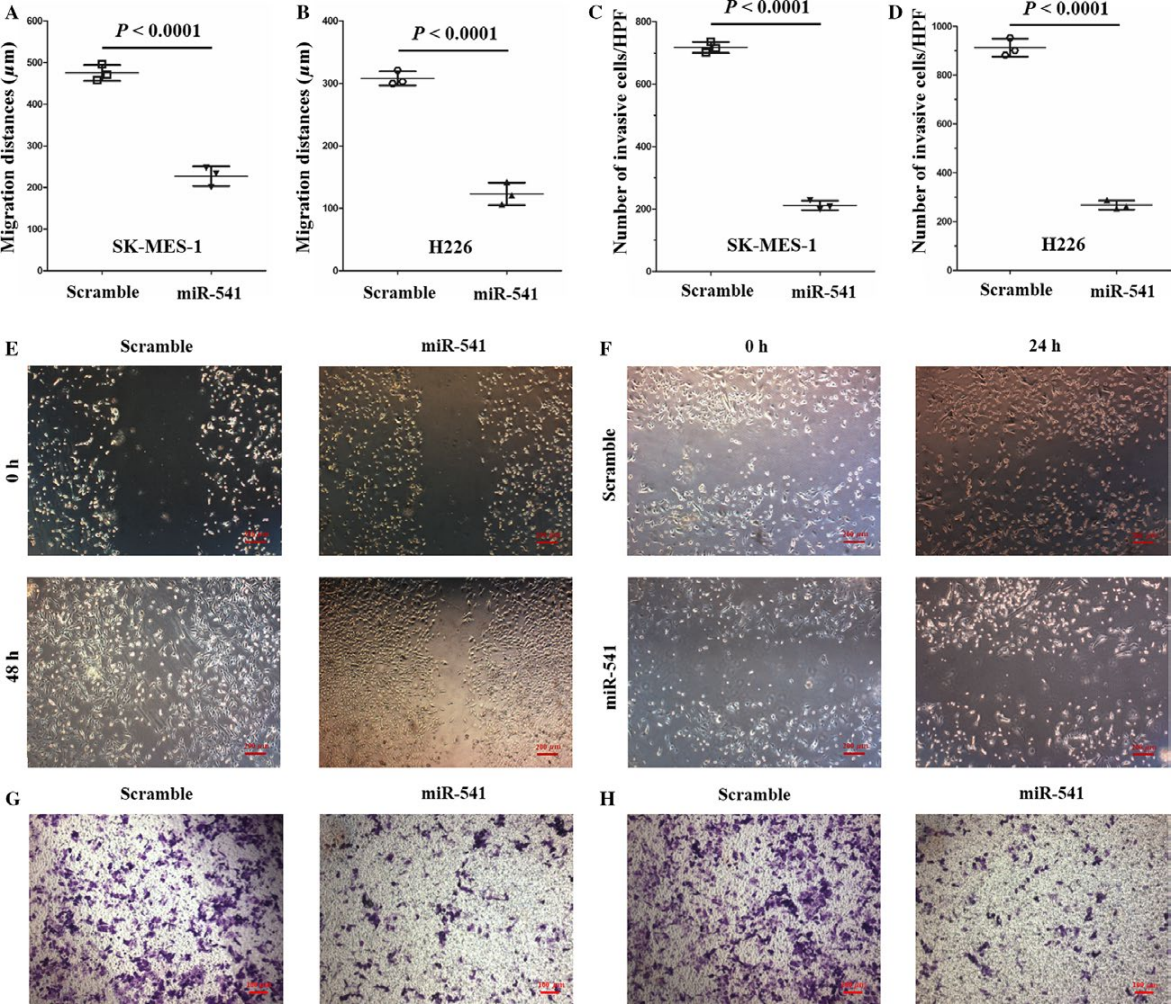
miR‐541 negatively regulated the migration and invasion abilities of SCLC cell lines. (A, E) Compared with the control cells, the miR‐541‐overexpressing SK‐MES‐1 cells demonstrated a significantly lower migration capability. The migration distance (*μ*m) of the SK‐MES‐1 cells was quantified (*n* = 3 for each cell group). (B, F) Compared with the control H226 cells, the miR‐541‐overexpressing H226 cells presented with a lower migration ability. The migration distance (*μ*m) of the H226 cells was quantified (*n* = 3 for each cell group). (C, G) The results of the transwell invasion assay revealed that the invasion ability was significantly decreased in the miR‐541‐overexpressing SK‐MES‐1 cells. The quantified data are presented as the number of invading cells per HPF. For each cell group, *n* = 3. (D, H) According to the transwell invasion results, miR‐541‐overexpressing H226 cells also demonstrated a reduced invasion capacity. The quantified data are presented as the number of invading cells per HPF (*n* = 3 for each cell group). HPF, high‐power field.

Before now, the number of studies that have investigated miR‐541 function is relatively few. A few studies have previously focused on miR‐541 function in the cardiovascular system. According to those studies, miR‐541 plays an important role in vascular smooth muscle cell proliferation and cardiac hypertrophy 19, 20. To explore miR‐541 function in SCLC, an in vitro cell model was established in our study through transfecting SK‐MES‐1 and H226 cells with miR‐541‐mimics. Cells transfected with miR‐541‐scramble served as controls. The miR‐541 expression levels were examined 36 h after transfection. The relative miR‐541 levels were increased 233.93‐fold and 148.50‐fold in the miR‐541‐overexpressing SK‐MES‐1 and H226 cells, respectively, compared with the control cells (Fig. 1C and D). As miR‐541 regulates cell proliferation in the cardiovascular system, the influence of miR‐541 expression on SK‐MES‐1 and H226 cell proliferation was examined using a CCK8 assay after the cells were transfected with the miR‐541‐mimics. miR‐541‐overexpressing SK‐MES‐1 and H226 cells presented with a decreased proliferation capacity at 24, 48, and 72 h posttransfection (Fig. 1E and F). Next, the wound scratch and transwell invasion chamber assays were applied to assess the impact of miR‐541 expression on the migration and invasion abilities of SK‐MES‐1 and H226 cells. The results revealed that compared with the control cells, the miR‐541‐overexpressing SK‐MES‐1 and H226 cells presented with decreased migration and invasion abilities (Fig. 2A–H). These results indicated that miR‐541 expression negatively regulates SK‐MES‐1 and H226 cell proliferation and invasion abilities.

### HMGA2 is a direct target of miR‐541 for repressing SCLC cell proliferation and invasion

**Figure 3:**
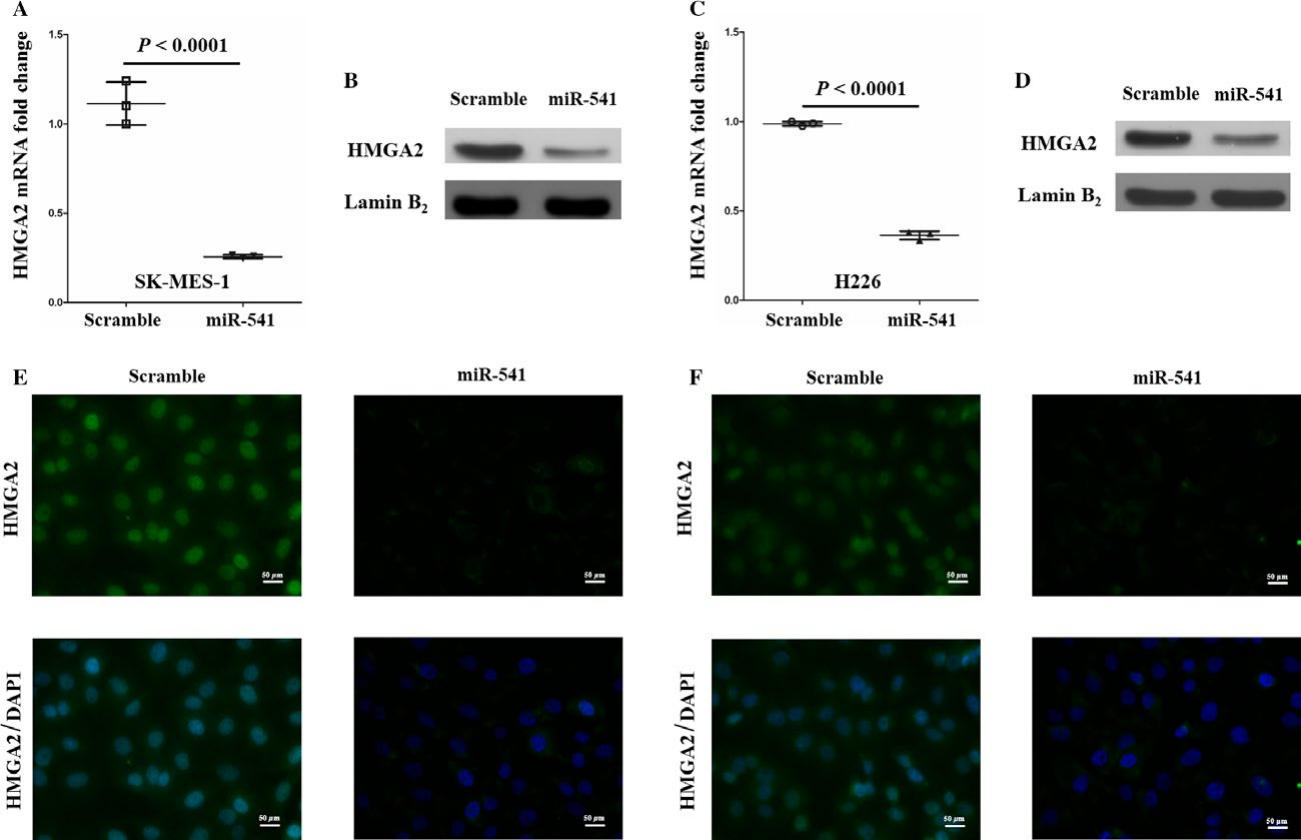
HMGA2 is a target of miR‐541 in SCLC cell lines. (A, C) The HMGA2 mRNA expression levels were significantly inhibited by miR‐541 overexpression in SK‐MES‐1 and H226 cells (*n* = 3 for each cell group). (B, D) The HMGA2 protein levels were significantly downregulated in the miR‐541‐overexpressing SK‐MES‐1 and H226 cells. Lamin B_2_ served as the internal control. A bigger field scan of the blot is shown in Fig. S1. (E, F) The immunocytochemistry results revealed that HMGA2 expression levels were decreased in the nucleus of miR‐541‐overexpressing SK‐MES‐1 and H226 cells.

**Figure 4:**
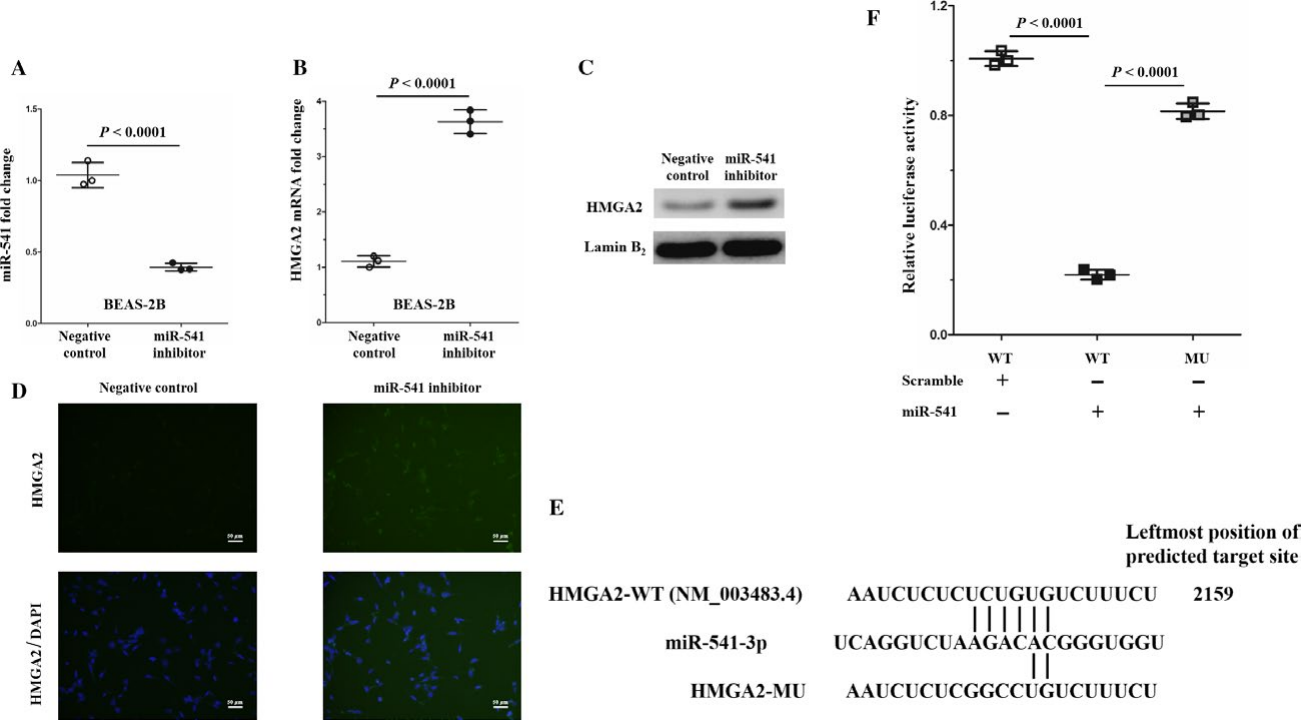
HMGA2 expression is upregulated in BEAS‐2B induced by miR‐541 inhibitor, and HMGA2 is a direct target of miR‐541. (A) Successful miR‐541 inhibition in BEAS‐2B cells induced by miR‐541 inhibitor. Cells transfected with negative control inhibitor served as the control cells (*n* = 3 for each cell group). (B) The HMGA2 mRNA expression levels were significantly enhanced by miR‐541 inhibition in BEAS‐2B cells (*n* = 3 for each cell group). (C) The HMGA2 protein levels were upregulated in the miR‐541‐inhibiting BEAS‐2B cells. Lamin B_2_ served as the internal control. A bigger field scan of the blot is shown in Fig. S1. (D) HMGA2 expression levels were enhanced in the nucleus of miR‐541‐inhibiting BEAS‐2B cells. (E) The RNA22 tool was used to predict that HMGA2 is a target gene of miR‐541. The predicted target site was on the 3′ UTR of HMGA2 mRNA. (F) Dual‐luciferase reporter assay analysis of the effect of miR‐541 expression on the 3′ UTR activity of HMGA2 mRNA in 293T cells (*n* = 3 experiments for all tests).

According to the aforementioned results, miR‐541 might be a tumor suppressor in SCLC. However, the molecular mechanism by which miR‐541 inhibits the proliferation and invasion of SK‐MES‐1 and H226 cells remains unclear. Commonly, miRNAs regulate the posttranscriptional expression of target genes through specifically binding the mRNA of target genes. Interestingly, we found that HMGA2 expression was decreased at both the mRNA and protein levels following the miR‐541‐mimics transfection in the SK‐MES‐1 and H226 cells (Fig. 3A–F). These findings indicated that miR‐541 might negatively regulate HMGA2 expression. To verify the regulation of miR‐541 on HMGA2, we performed loss‐of‐function experiments through transfecting BEAS‐2B cells with miR‐541 inhibitor. Cells transfected with negative control inhibitor served as controls. The miR‐541 expression levels were examined 36 h after transfection. The relative miR‐541 levels were significantly decreased in the miR‐541‐inhibiting BEAS‐2B cells (Fig. 4A). Next, we found that HMGA2 expression was increased at both the mRNA and protein levels following the miR‐541 inhibitor transfection in the BEAS‐2B cells (Fig. 4B–D). Taken together, these evidences strongly suggested that miR‐541 negatively regulated HMGA2 expression.

HMGA2, a member of the chromatin remodeler family, is highly expressed in several human malignant tumors and is considered to be an oncogene according to previous studies 21, 22. Our recent study demonstrated that HMGA2 is an oncogene in SCLC 13. As HMGA2 expression was negatively regulated by miR‐541, we hypothesized that HMGA2 might be a direct target of miR‐541. Using the RNA22 tool (cm.jefferson.edu/rna22/Interactive version 2.0), we found a potential target site for miR‐541 on the 3′ UTR of HMGA2 mRNA (Fig. 4E). To identify whether this potential binding site is a miRNA‐response element, we used QuikChange PCR to obliterate this potential binding site on the HMGA2 mRNA 3′ UTR (Fig. 4E). Next, we constructed two luciferase reporter vector recombinant plasmids, psiCHECK‐HMGA2‐WT and psiCHECK‐HMGA2‐MU, via inserting the 3′ UTR of HMGA2 mRNA. We found that mutations at the potential site strongly reversed the decreased luciferase activity induced by miR‐541 overexpression (Fig. 4F). These results, for the first time, revealed that HMGA2 is a direct target of miR‐541.

**Figure 5:**
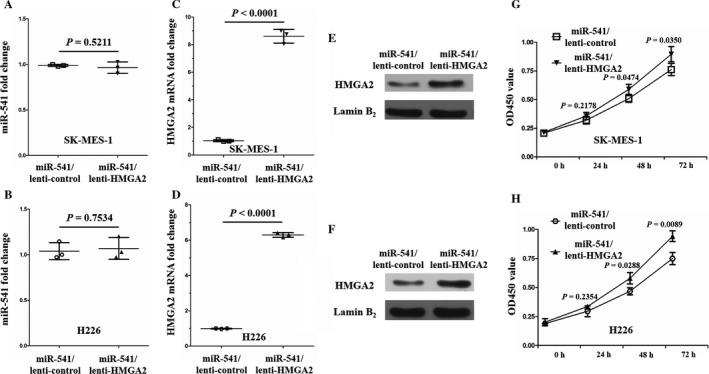
Successful HMGA2 overexpression in miR‐541‐overexpressing SK‐MES‐1 and H226 cells, and the enhanced proliferation of miR‐541‐overexpressing SK‐MES‐1 and H226 cells by HMGA2 overexpression. (A) The difference in the miR‐541 expression levels between the miR‐541/lenti‐HMGA2 and miR‐541/lenti‐control SK‐MES‐1 cells was not significant (*n* = 3 for each cell group). (B) HMGA2 overexpression in miR‐541‐overexpressing H226 cell also did not significantly change miR‐541 expression (*n* = 3 for each cell group). (C, D) Verification of HMGA2 mRNA overexpression in miR‐541‐overexpressing SK‐MES‐1 and H226 cells (*n* = 3 for each cell group). (E, F) The HMGA2 protein levels were significantly upregulated in the miR‐541/lenti‐HMGA2 SK‐MES‐1 and H226 cells. Lamin B_2_ served as the internal control. A bigger field scan of the blot is shown in Fig. S1. (G, H) Higher OD450 values were observed in the miR‐541/lenti‐HMGA2 SK‐MES‐1 and H226 cells than in the control cells at 48 and 72 h (*n* = 3 for each cell group). OD450, optical density at 450 nm.

**Figure 6:**
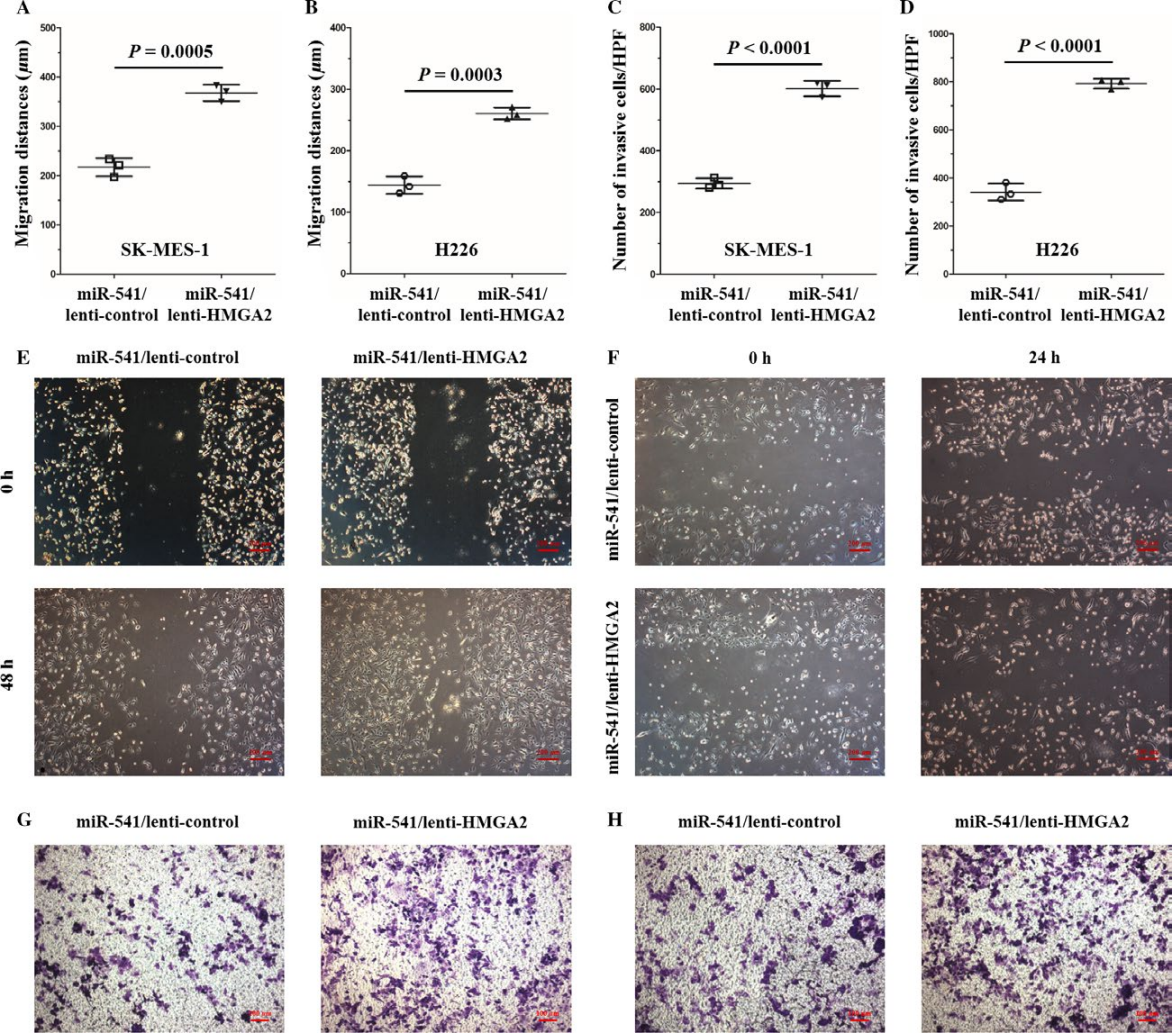
HMGA2 overexpression weakened the inhibitory effects of miR‐541 on the migration and invasion capabilities of the SCLC cell lines. (A, E) The migration capacity was significantly higher in the miR‐541/lenti‐HMGA2 SK‐MES‐1 cells than in the control cells. The migration distance (*μ*m) of the SK‐MES‐1 cells was quantified (*n* = 3 for each cell group). (B, F) Compared with the control cells, the miR‐541/lenti‐HMGA2 H226 cells presented with a higher migration ability. The migration distance (*μ*m) of the H226 cells was quantified (*n* = 3 for each cell group). (C, G) The transwell invasion assay results revealed that invasion ability was significantly increased in the miR‐541/lenti‐HMGA2 SK‐MES‐1 cells. The quantified data are presented as the number of invading cells per HPF (*n* = 3 for each cell group). (D, H) The miR‐541/lenti‐HMGA2 H226 cells also demonstrated a higher invasion capacity. The quantified data are presented as the number of invading cells per HPF (*n* = 3 for each cell group). HPF, high‐power field.

HMGA2, an acknowledged oncogene by several previous studies 21, 22, is involved in SK‐MES‐1 cell proliferation and invasion according to our previous study 13. The finding that HMGA2 is a direct target of miR‐541 made us propose a new hypothesis: miR‐541 represses SK‐MES‐1 and H226 cell proliferation and invasion via directly inhibiting HMGA2 expression. To test this hypothesis, we cotransfected SCLC cells (SK‐MES‐1 and H226) with miR‐541‐mimics and lenti‐HMGA2 (miR‐541/lenti‐HMGA2 cells), and the control cells were cotransfected with miR‐541 and lenti‐control (miR‐541/lenti‐control cells). Thirty‐six hours after transfection, the miR‐541 and HMGA2 expression levels were examined in the miR‐541/lenti‐HMGA2 SK‐MES‐1 and H226 cells and in the miR‐541/lenti‐control cells. The difference in miR‐541 expression between the experimental and control cells was not significant (Fig. 5A and B). However, the HMGA2 mRNA and protein expression levels were both significantly higher in the experimental cells than in the control cells (Fig. 5C–F). Next, the proliferation capacity of the experimental and control cells was examined. According to the results, the experimental cells presented with an increased proliferation ability at 48 and 72 h after transfection (Fig. 5G and H). Regarding the wound scratch and transwell invasion assay results, the experimental cells also presented with increased migration and invasion capabilities (Fig. 6A–H). Taken together, these results suggested that miR‐541 represses the proliferation and invasion of SK‐MES‐1 and H226 cells via directly targeting HMGA2.

## Discussion

In this study, we reported that miR‐541 expression was downregulated in SCLC cancerous tissue compared with adjacent normal tissue. We also found that miR‐541 inhibited the proliferation and invasion of SCLC cells. Moreover, miR‐541 inhibited SCLC cell proliferation and invasion via directly negatively regulating HMGA2 expression, which is an important protein that can promote cancer progression and metastasis 23, 24, 25. Based on these results, we concluded that miR‐541 might act as a tumor suppressor in SCLC.

Many miRNAs are deeply implicated at multiple steps of tumor diseases, including proliferation, invasion, adhesion, and metastasis 7, 8, 26. Currently, only a few studies have reported the function of miR‐541 in oncology. A previous study revealed the tumor‐suppressing role of miR‐541 in breast cancer 16. According to Lu and colleagues, miR‐541 expression is decreased in lung cancer 18. However, the functions of miR‐541 in cardiovascular diseases are better understood. Liu and colleagues reported that an interaction between angiotensin II and miR‐541 regulates cardiac hypertrophy 20. Another study revealed that miR‐541 is involved in regulating vascular smooth muscle cell proliferation 19. In the present study, our results indicating that miR‐541 expression is decreased in SCLC are consistent with the findings by Lu 18. In addition, the finding that miR‐541 is a tumor suppressor in both breast cancer and SCLC makes miR‐541 potentially valuable in the clinical treatment of human tumor diseases.

HMGA2 is a member of the nonhistone chromosomal high‐mobility group protein family. As a member of the HMGA family of architectural transcription factors, HMGA2 affects DNA‐dependent activities and influences cell growth, proliferation, differentiation, and death by modifying the structure of binding partners to generate a conformational change 27. In general, HMGA2 is normally highly expressed during embryonic development but maintains a low expression level in tissue after adulthood. However, HMGA2 is reactivated in many malignancies, such as leukemia, gastric cancer, ovarian cancer, and NSCLC 21. HMGA2 functions have been studied extensively, and an increasing number of studies acknowledge HMGA2 as an oncogene. Li and colleagues found that HMGA2 enhanced epithelial–mesenchymal transition (EMT) in colon cancer by inducing the transcription factor slug 22. According to our previous study, ANG‐activated HMGA2 enhances SCLC progression 13. Yang and colleagues found that PAR1/HMGA2 pathway positively regulates invasion of breast cancer cell 28. According to Haselmann V and colleagues, TRAIL‐R2 promotes proliferation of pancreatic cancer cell lines via inhibiting let‐7/HMGA2 pathway 29. However, serval studies have also highlighted the negative regulation of HMGA2 expression by miRNAs in malignancies 30, 31. In this study, to explore the mechanism by which miR‐541 inhibits SK‐MES‐1 and H226 cell proliferation and invasion, we used luciferase assays and rescue experiments, and for the first time, we successfully demonstrated that HMGA2 is a direct target of miR‐541. Our findings enrich the miRNA regulatory network of HMGA2 in malignancies.

In summary, the results in our study all confirm that miR‐541 functions as a tumor suppressor in SCLC by targeting HMGA2. However, further relevant in vivo experiments that confirm the potential value of miR‐541 for clinical SCLC treatment and that investigate other mechanisms by which miR‐541 suppresses tumors are still needed.

## Ethics Statement

All protocols in this study were approved by the Institutional Review Board of Chengdu Military General Hospital (Chengdu, People's Republic of China). Written informed consent was gained from all patients with SCLC for examinations utilizing human lung specimens.

## Conflict of Interest

None declared.

## Supporting information


**Figure S1.** A, B. Western blot showing suppressed levels of HMGA2 resulting from miR‐541 overexpression in SCLC cell lines. C, D. Western blot showing increased expression levels of HMGA2 in miR‐541/lenti‐HMGA2 SCLC cell lines. E. Enhanced expression level of HMGA2 protein resulting from miR‐541 inhibition in BEAS‐2B cell.
