# Supplementary figures and images for: miR‐541 suppresses proliferation and invasion of squamous cell lung carcinoma cell lines via directly targeting high‐mobility group AT‐hook 2
Source: Cancer Med. 2018 Apr 16;7(6):2581–91. doi: 10.1002/cam4.1491 (PMC6010725; doi:10.1002/cam4.1491)

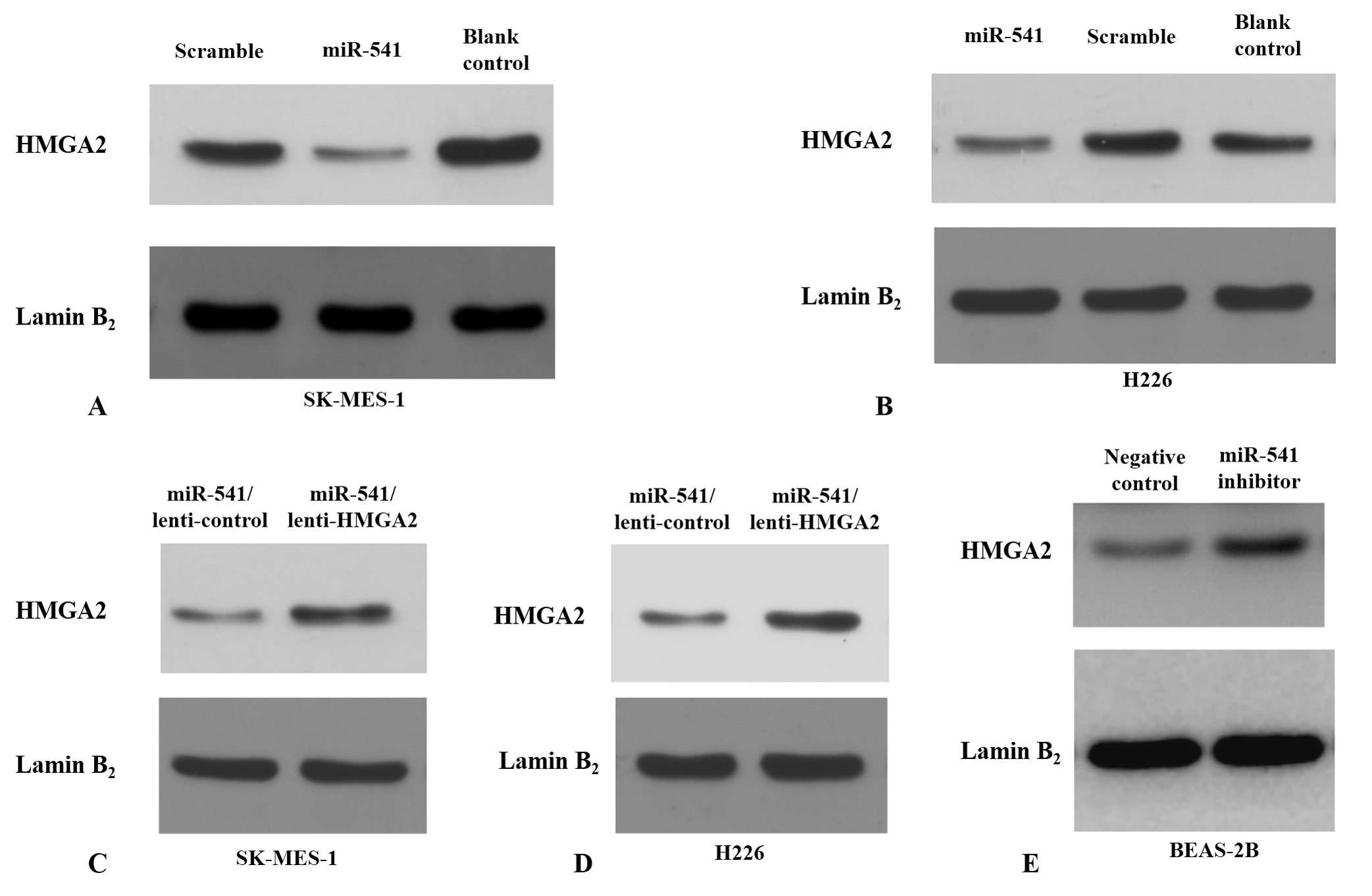

Supplement: Supplementary file 1 — Figure S1. A, B. Western blot showing suppressed levels of HMGA2 resulting from miR‐541 overexpression in SCLC cell lines. C, D. Western blot showing increased expression levels of HMGA2 in miR‐541/lenti‐HMGA2 SCLC cell lines. E. Enhanced expression level of HMGA2 protein resulting from miR‐541 inhibition in BEAS‐2B cell. [file CAM4-7-2581-s001.tif]
